# Lack of Objective Measurement in the Initial Screening and Follow-Up of Patients Who Report Whiplash Injury—Is Elastography of the Trapezius Muscle an Answer?

**DOI:** 10.3390/jcm11133851

**Published:** 2022-07-02

**Authors:** Jure Aljinović, Blaž Barun, Benjamin Benzon, Ana Poljičanin, Tonko Vlak

**Affiliations:** 1 Institute of Physical Medicine and Rehabilitation with Rheumatology, University Hospital Split, Šoltanska 1, 21000 Split, Croatia; blaz.barun1@gmail.com (B.B.); ana.poljicanin@gmail.com (A.P.); tonkovlak@gmail.com (T.V.); 2 Department for Health Studies, University of Split, 21000 Split, Croatia; 3 Departments of Anatomy, Histology and Embryology, School of Medicine, University of Split, 21000 Split, Croatia; benzon.benjamin@gmail.com; 4 Department of Physical and Rehabilitation Medicine, School of Medicine, University of Split, 21000 Split, Croatia

**Keywords:** whiplash injury, elastography, ultrasound, stiffness, trapezius, shear wave

## Abstract

Background: Painfully decreased cervical range of motion accompanied by muscle spasm is a common presentation of whiplash injury of the neck. Stiffness of the cervical muscles can be assessed by ultrasound shear wave elastography (SWE), expressed in kilopascals (kPa). The hypothesis: SWE of the trapezius muscle is an objective measurement suitable for the initial screening and follow-up of patients who report whiplash injury. Methods and results: A total of 99 patients after whiplash injury were compared to 75 control participants. Mean trapezius stiffness was 82.24 ± 21.11 vs. 57.47 ± 13.82 for whiplash patients and controls, respectively. The cut-off value of SWE of 75.8 kPa showed 77% accuracy in correctly assigning patients to the whiplash or control group. To evaluate whether SWE can be used as a follow-up method of recovery after a whiplash injury, initial and endpoint SWE (after six months, *n* = 24) was carried out. Patients reporting no recovery showed similar SWE values as completely recovered patients. This finding refutes the second part of our hypothesis. Conclusions: SWE is a method that can be used for the initial screening of patients with whiplash injury, but we are still searching for an objective measurement that can be used in the follow-up of recovery.

## 1. Introduction

Whiplash injury of the neck is very common after “non-catastrophic” traffic accidents, with an incidence of more than 300 persons per 100,000 people [1], and it is defined as a bony or soft-tissue injury caused by sudden acceleration/deceleration of the head and neck [2]. The diagnosis is based on the mechanism of the injury. Patient-reported symptoms can be multiple and diverse (headache, paresthesia, pain, spasm, weakness, etc.) and are generally classified as whiplash-associated disorder (WAD) [3]. Painfully restricted cervical range of motion accompanied by muscle spasm is a common presentation in patients with whiplash injury [2]. Prolonged sick leaves and lower productivity after returning to work after the injury due to chronic pain and disability significantly impact the healthcare system [4].

In WAD, patients can report a wide variety of whiplash-related symptoms, and there are no standardized objective measurements to confirm or dismiss them. That creates the space for insurance claims seeking financial compensation [5]. The assessment of the severity and longevity of the disability after the injury is acquired by patient-reported indices and can add to potential malingering, estimated at 15% to 40% [6].

Three components of whiplash injury need to be analyzed for eventual objective measurement: pain, range of motion, and muscle spasm. Modern medicine does not possess a quantitative tool to measure other people’s pain level. Pain levels are obtained through patient-reported indices, most commonly the visual analog scale (VAS) or numerical rating scale (NRS). Multidimensional pain questionnaires are rarely used in everyday practice.

Cervical range of motion (CROM) after the injury can be measured with a goniometer. It is an accurate and valid measurement often used by physiotherapists in everyday practice. CROM restriction is associated with pain level [7], and both correlate with the severity of the injury, but only to be dismissed as a method of follow-up [8]. The main reason is that their values can vary in a day. For example, if the patient wakes up with only slight pain in the neck, but tries to read newspapers, one can aggravate the pain for the whole day. In addition, both measurements are susceptible to potential malingering either by reporting worse possible pain or unwillingness to do the maximal possible movement of the cervical spine.

Increased stiffness or spasm of the cervical muscles is the third category that needs to be discussed. Increased muscle spasm of the cervical region is reported by almost all patients with a whiplash injury [3]. It can be a normal evolutionary mechanism for the prevention of further injuries, but its prolonged state can enhance disability after the injury. Palpation of the trapezius muscles is an integral part of the clinical examination to assess whether the affection is symmetrical or one side is more affected. When all of this is put into perspective, a need for objective measurement of muscle stiffness is crucial.

Ultrasound shear wave elastography (SWE) is a method that can assess the qualitative and quantitative elasticity properties of soft tissues, including the muscles. Tissue stiffness is measured by shear modulus and expressed in pressure units (kilopascals (kPa)). The SWE method uses a combination of the radiation force induced in tissue by an ultrasonic beam and the ultrafast imaging sequence capable of catching the propagation of the resulting shear waves in real time [9].

This procedure is safe, reproducible, and does not involve any radiation for the patient or examiner. It is also not time consuming when performed by an experienced radiologist. When using SWE, all participants are instructed to relax the shoulder girdle, and SWE is performed after confirmation of no muscle contraction in the B-mode image. Potential pitfalls of this method are that the standardization of the examination procedure is required, along with more radiologists versed in elastography measurement, more ultrasound machines with an elastography mode, and finally, the values of the stiffness of the cervical muscles in the general population subdivided by age and gender.

### 1.1. Hypothesis of This Review

Ultrasound shear wave elastography of the trapezius muscle is an objective measurement suitable for the initial screening and follow-up of patients who report a whiplash injury.

### 1.2. Evaluation of the Hypothesis

Our group of authors previously published the results for the stiffness of different muscles in the neck region after a whiplash injury. Seventy-five people with whiplash injuries were compared with 75 age- and gender-matched controls. The results showed that sternocleidomastoid and splenius capitis muscle SWE could not be used in diagnosing whiplash injury due to the highly asymmetrical data distribution and variance in tone of 300% between patients [10]. Trapezius muscle showed symmetrical data distribution with clinically relevant results described by higher stiffness in whiplash injury than in the control population group (87.84 ± 23.23 kPa vs. 57.47 ± 13.82 kPa, *n* = 75). There was no statistical difference between the left and right trapezius, which proved symmetrical affection of the neck.

To evaluate this hypothesis, we included an additional 24 patients after a whiplash injury (*n* = 99). The mean trapezius stiffness was 82.24 ± 21.11 kPa; the patients were aged 41.9 ± 13.

Knowing only the SWE value of the participant, a classification model using logistic regression was calculated (n-whiplash = 99, n-control = 75). The cut-off value of SWE of 75.8 kPa showed 77% accuracy in correctly assigning patients either to the whiplash or the control group (94.7% specificity; 63.6% sensitivity, *p* < 0.0001, ROC (AUC, area under curve) = 0.86).

When the classification model was calculated using SWE value, age, and gender, an accuracy of 82.7% was obtained (93.3% specificity; 74.7% sensitivity, *p* < 0.0001, AIC evidence ratio = 1108 when compared to SWE only model, ROC (AUC) = 0.89, probability cut-off of at 65%).

**Figure 1:**
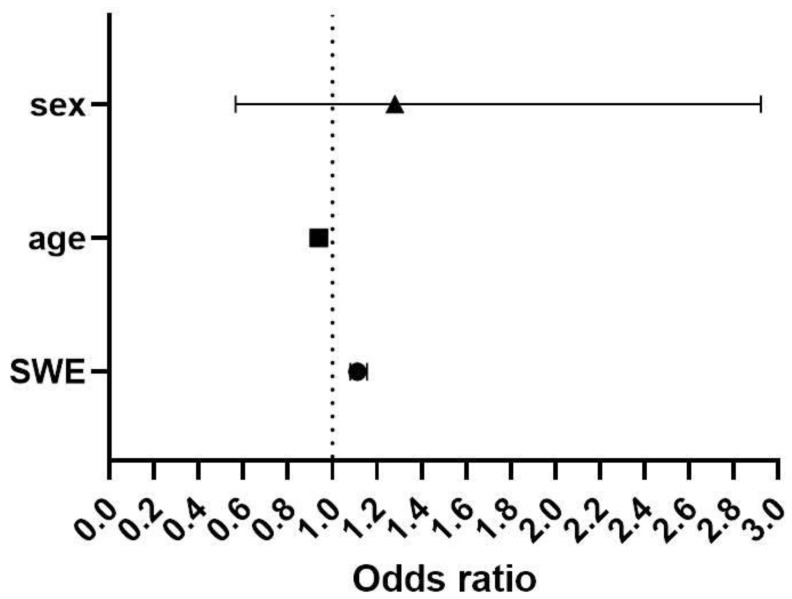
Likelihood of occurrence of whiplash injury in response to age, gender, and SWE. $probability\;of\;injury\;\left(\%\right)=\frac{1}{1+e^{4.326-0.017\cdot SWE-0.248\cdot sex+0.063\cdot age}}$. Sex = 0 for male and 1 for female, SWE should be expressed in kPa and age in years.

In Figure 1, the effects of age, gender, and SWE values on the occurrence of whiplash injury were calculated. An increase in SWE value by 1 kPa is associated with a 1.12-fold increase in the odds of whiplash injury (OR = 1.12, 95% confidence interval (CI) of 1.08–1.15). Older age lowered the odds of whiplash injury for each year 0.93-fold (OR = 0.93, 95% CI 0.9–0.96). No conclusion can be made regarding gender’s effect on the occurrence of whiplash injury. The female gender showed an OR of 1.2 (95% CI 0.56–2.92), and further studies are needed to explain such width in an estimate of effect.

This finding gives us positive evidence for the initial part of our hypothesis: SWE can be used in the screening of whiplash injury with the cut-off value of 75.8 kPa, which can be used to correctly assign three out of four patients in the control or whiplash group by knowing only the SWE.

To evaluate the second part of our hypothesis, that SWE can be used for the follow-up of whiplash injury patients, we carried out additional research.

Our group of authors reported SWE measurements after whiplash injury at baseline and after six months. The measurements were taken by two radiologists, and a decrease in stiffness was reported by both (first ∆10.1 kPa; *p* = 0.04; second ∆8.63 kPa; *p* = 0.07). In addition, excellent intra- and inter-observer reliability was reported [11].

However, when we compared the endpoint SWE values with the patient-perceived recovery values, we found no difference in the SWE values between the recovered and non-recovered patients after six months (55.6 ± 9.7 vs. 57 ± 15.8, ∆1.45; *p* = 0.82).

This finding refutes the second part of our hypothesis, that SWE can be used as a follow-up tool after a whiplash injury. Although a decrease in trapezius stiffness was detected, the correlation with patient-reported recovery was not found, so patients reporting no recovery showed similar SWE values as completely recovered patients.

The trapezius tone one year after a whiplash injury is still unknown.

## 2. Discussion

Most patients, after a whiplash injury, have symptoms without radiologically apparent bony or soft tissue injury. Therefore, imaging (X-ray, CT, or MRI) initially used to evaluate patients cannot be used to determine the seriousness of the injury. However, the value of MRI as a prognostic factor for functional and neurological outcomes was described in cervical subaxial spinal cord injuries [12]. Even though the evidence is still inconclusive [13,14], some recent studies showed a correlation between preexisting facet joint degeneration or constitutional cervical sagittal alignment (low neck tilt and low thoracic inlet angle) on CT and worse recovery [15,16]. Further studies are needed to confirm the usefulness of those radiologic findings in predicting outcomes.

Our hypothesis focuses on a prolonged spasm of the cervical muscles as a possible reason for pain and disability after a whiplash injury. Painful muscle spasm is a symptom present in every patient with a whiplash injury of the neck. Although the literature does not support the effectiveness of muscle relaxants in whiplash, their short-term use is common in everyday practice [17]. Sometimes, in the interview with the patients, we find that using a muscle relaxant, like diazepam, has more effect on alleviating the symptoms than using analgesics or non-steroid anti-inflammatory drugs. Physical medicine interventions aimed at decreasing the spasm of the muscles, like electrotherapy or ultrasound therapy, are commonly used in clinical practice, but there is no evidence to support the benefit of their use [18].

SWE was used as a method for the evaluation of our hypothesis. It is a new diagnostic method for this purpose, and it requires standardization (e.g., positioning of the patient or number of measurements). Ultrasound examination can detect the voluntary contraction of the muscles in B-mode, which is an advantage of this method. If contraction is detected, the patient is advised to relax the muscles. We assume this can prevent people from voluntarily trying to increase muscle stiffness.

The initial part of the hypothesis, that SWE can be used to diagnose whiplash injury, was confirmed, since increased stiffness in the trapezius muscle was detected in the whiplash injury group compared to the control group. We also calculated the classification model that correctly assigns patients to the whiplash or control group in 77% of the cases only by knowing the SWE value (cut-off value 75.8 kPa).

Our data showed that six months after the whiplash injury, most patients had decreased trapezius muscle stiffness of around 10 kPa. However, similar values of muscle stiffness were found in both patients who reported complete recovery and no recovery. Therefore, this value is not clinically relevant and cannot support the second part of our hypothesis that SWE can be used as a follow-up method. We presume that physical therapy, which included medical exercise, transcutaneous electric nerve stimulation (TENS, as a form of analgesic electro procedure), and therapeutic ultrasound of cervical muscle groups, provided a decrease in muscle stiffness in all patients after the injury. However, when performing activities of daily living (ADL) such as reading, cooking, or driving, adequate strength of the neck muscle groups is required to maintain the neck in a fixed position for an extended period of time. These activities can induce pain and cannot be detected by one-dimensional diagnostics like stiffness evaluation by SWE. We assume that an increase in the adherence to exercise after the institutionalized PT and progressive strengthening of the neck muscle groups can provide more extended painless periods in ADLs and increase the quality of life, and future studies could answer this hypothesis. The decrease in muscle stiffness after PT is only a component of recovery, which is influenced by other physical and psychological factors. Unlike SWE, patient-reported outcome measurements that assess all parts of human functioning through one-day, vocational, avocational, and everyday activity, like the Neck Disability Index (NDI), are used as follow-up tools of disability after a whiplash injury [19]. Given that the NDI is a subjective tool, patients can use it to aggravate the level of disability when seeking financial compensation. The NDI was previously validated and used by our group of authors and its superiority to SWE in the follow-up of whiplash injury patients has been confirmed [11,20].

To conclude, SWE is a method that can be used for the initial screening of patients who report whiplash injury, but we are still searching for an objective measurement that can be used in the follow-up of recovery after a whiplash injury.

## Data Availability

Data are available upon request.
